# Adequate 25(OH)D moderates the relationship between dietary inflammatory potential and cardiovascular health risk during the second trimester of pregnancy

**DOI:** 10.3389/fnut.2022.952652

**Published:** 2022-07-29

**Authors:** Wan-jun Yin, Li-jun Yu, Lin Wu, Lei Zhang, Qiong Li, Fei-cai Dai, Rui-xue Tao, Xiao-min Jiang, Peng Zhu

**Affiliations:** ^1^Department of Maternal, Child and Adolescent Health, School of Public Health, Anhui Medical University, Hefei, China; ^2^MOE Key Laboratory of Population Health Across Life Cycle, Hefei, China; ^3^NHC Key Laboratory of Study on Abnormal Gametes and Reproductive Tract, Anhui Medical University, Hefei, China; ^4^Anhui Provincial Key Laboratory of Population Health and Aristogenics, Anhui Medical University, Hefei, China; ^5^Department of Gynecology and Obstetrics, Hefei First People’s Hospital, Hefei, China; ^6^Department of Obstetrics and Gynecology, Anhui Province Maternity and Child Health Hospital, Hefei, China

**Keywords:** vitamin D, cardiovascular health, pregnant women, dietary inflammatory potential, nutrients

## Abstract

**Background:**

Pro-inflammatory diets play an important role in developing cardiovascular disease (CVD). Vitamin D has been demonstrated to have an anti-inflammatory effect and promote cardiovascular health (CVH). However, it is unclear whether adequate vitamin D during pregnancy protects against poor CVH caused by pro-inflammatory diets.

**Objective:**

To investigate the association of pro-inflammatory diets with the cardiovascular risk (CVR) among pregnant women and whether such association was modified by vitamin D status.

**Methods:**

The study was based on a prospective birth cohort that included 3,713 pregnant women between 16 and 23 gestational weeks. In total, 25(OH)D concentrations and high-sensitivity C-reactive protein (hs-CRP) were measured from the collected blood. The dietary inflammatory potential was evaluated using the empirical dietary inflammatory pattern (EDIP) score based on a validated food frequency questionnaire. Gestational CVR was evaluated using the CVR score based on five “clinical” CVR metrics, including body mass index, blood pressure, total cholesterol, glucose levels, and smoking status.

**Results:**

The proportion of women with a CVR score >0 was 54.3%. We observed a positive association between the EDIP score and CVR score. Compared with the lowest quartile, the CVR score (β = −0.114, 95% CI, −0.217, −0.011) and hs-CRP levels (β = −0.280, 95% CI, −0.495, −0.065) were lower in the highest quartile (*P* for trend <0.05). Increased CVR connected with high EDIP score was observed only in women with 25(OH)D concentrations <50 nmol/L (RR = 1.85; 95% CI: 1.35, 2.54). Mediation analysis revealed that the proportion of association between the EDIP score and CVR score mediated by 25(OH)D was 28.7%, and the proportion of the association between 25(OH)D and the CVR score mediated by hs-CRP was 21.9%.

**Conclusion:**

The higher dietary inflammatory potential was associated with an increased CVR during pregnancy by promoting inflammation. Adequate vitamin D could exert anti-inflammatory effects and modify such association.

## Introduction

Cardiovascular disease (CVD) accounted for 40% of the deaths and is the leading cause of death and premature death in China (1). Pregnancy poses an immense challenge to women’s metabolic function and cardiometabolic stressors and is more susceptible to cardiovascular damage (2). Recent evidence suggests that the mother’s cardiovascular health (CVH) during pregnancy was significantly associated with the later cardiometabolic health among women and offspring (3).

Inflammation has been implicated in CVD etiology (4), and increasing inflammation may lead to a poor gestational CVH. The higher dietary inflammatory potential that leads to increased inflammation levels was associated with a higher risk of CVD (5). A diet intervention study found that high-inflammation levels moderated the effects of a diet intervention to control CVD (6, 7). Thus, interventions to reduce inflammation and thus protect CVH applicable to pregnant women are required, and vitamin D supplementation is an attractive target.

Vitamin D can regulate inflammation and is generally deficient during pregnancy (8). Previous intervention experiments have demonstrated that daily vitamin D supplementation will decrease systemic inflammatory markers such as high-sensitivity C-reactive protein (hs-CRP) (9). A similar relationship was found in our earlier study of high serum 25(OH)D concentrations during pregnancy which were inversely related to hs-CRP levels (10). Moreover, a recent large-scale population meta-analysis has confirmed that a low vitamin D level increases CVD risk (11). These studies indicate that vitamin D may inhibit inflammation and promote gestational CVH. So far, however, there has been a little discussion about whether adequate 25(OH)D moderates the relationship between dietary inflammatory potential and cardiovascular risk (CVR) during pregnancy.

Therefore, in this study, we tested the relationship between dietary inflammatory potential and CVR during pregnancy and whether such a relationship was modified by 25(OH)D concentrations. It could conceivably be hypothesized that 1) high-dietary inflammatory potential was associated with increased CVR during pregnancy by promoting inflammation, and 2) adequate 25(OH)D concentrations may modify such association by inhibiting inflammation.

## Methods

### Study participants and design

The data of this study was from a prospective birth cohort study. In the cohort study, a total of 4,216 pregnant women aged 18 to 45 years, with gestational ages from 16 to 23 weeks, were recruited in three hospitals (The First Affiliated Hospital of Anhui Medical University, Anhui Women and Child Health Care Hospital, and The First People’s Hospital of Hefei City) from March 2018 and June 2021. The exclusion criteria included the following: missing blood samples, severe anemia, abnormal liver, renal, or thyroid function, ongoing infections (e.g., cervicovaginal infection and periodontal infection), and incomplete CVR data during pregnancy. In addition, pregnant women with hs-CRP concentrations >10 mg/L were excluded (12), as these likely indicate acute inflammatory response. Given that blood pressure (BP) is a component of gestational CVR, we did not exclude participants with eclampsia or pre-eclampsia.

At recruitment, weight and height were measured by well-trained staff using standardized procedures. The participants completed a structured questionnaire including sociodemographic characteristics, lifestyle, and perinatal health status through face-to-face interviews or medical records. Each participant completed a validated food frequency questionnaire (FFQ) at recruitment. Then, study nurses collected samples of the venous blood. At last, we obtained 3,713 pregnant women’s complete data, including blood samples (Supplementary Figure 1). The ethical approval was granted by the Ethics Committee of Anhui Medical University (20180092), and informed consent was obtained from each participant.

### Dietary assessment

The nutrition information of the participants was assessed using an FFQ at 16–23 gestational weeks, pregnant women’s self-reported food intake frequency, and serving size in the past month. Specified serving sizes are described by using natural portions (e.g., 1 tomato) or standard weight and volume measures of the servings commonly consumed (13). With responses ranging from “never” to “1 time a day or more”, answers followed: never = 0 times/day; one to two times a week = 0.2/day; three to six times a week = 0.6/day; more than once per day = 1/day.

### Assessment of the dietary inflammatory potential

The dietary inflammatory potential was assessed by the empirical dietary inflammatory pattern (EDIP) score. The development of the EDIP score was based on the previous studies (5, 14). It is based on circulating concentrations of 3 systemic inflammatory biomarkers, including interleukin-6, C-reactive protein (CRP), and tumor necrosis factor-α receptor 2 (TNFα-R2), to assess the overall subversive potential of diets. In brief, plasma levels of interleukin 6, TNFα-R2, and CRP were regressed on 39 pre-defined food groups by using reduced-rank regressions and stepwise linear regressions, selecting 18 food groups most predictive of these biomarkers. The EDIP was calculated as the weighted sum of these 18 food groups with weights (i.e., the contributions of each food to the overall score) equal to the coefficients from the stepwise regression. So, the food group with negative values suggests that these are anti-inflammatory foods. In this study, pizza was omitted because of the traditional Chinese-feeding habits. Therefore, dietary intakes of 17 food groups were used to calculate the EDIP score, including refined grains, processed meat, red meat, organ meat, other fish, other vegetables, high-energy beverage, low-energy beverages, tomatoes, organ meat, green leafy vegetables, fruit juice, beer wine, tea, coffee, snacks, and dark yellow vegetable. The EDIP calculation, including the average daily intake of each food group, was first divided by a specific group of servings (13) to determine its information; these values were then multiplied by its particular inflammatory coefficient (15) and compared to add, the final value is adjusted by dividing by 1,000 (Supplementary Table 1). The EDIP score was represented as pro-inflammatory diets with a higher score and anti-inflammatory diets with a lower score.

### Assessment of gestational cardiovascular risk

Gestational CVR was evaluated using the CVR score at 24 to 28 gestational weeks. The CVR score model can be an effective and straightforward tool for the cardiovascular disease forecasting and warning. The CVR score model was based on the five “clinical” CVR metrics (body mass index [BMI], BP, total cholesterol [TC] level, smoking status, and blood glucose level. Each CVR metric was classified as ideal (0 points), intermediate (1 point), or poor (2 points). Increased CVR was defined as more than 0 points. The detailed classification criteria are as follows: BMI (kg/m^2^): ideal: ≤28.4, intermediate: 28.5–32.9, poor: ≥33. BP (mmHg): ideal: systolic blood pressure (SBP) <120 and diastolic blood pressure (DBP) <80, intermediate: SBP 120-139, or DBP 80–89, poor: SBP ≥140 or DBP ≥90. TC (mg/dL): ideal: <260, intermediate: 260–299, poor: ≥300. Blood glucose (mg/dl): ideal: non-gestational diabetes mellitus (GDM), poor: GDM: fasting ≥92, 1-h oral glucose tolerance test (OGTT) ≥180, 2-h OGTT ≥ 153 (3). The results of four “clinical” CVR metrics (BMI, BP, TC, and blood glucose) were obtained from the hospitals at 24 to 28 gestational weeks. Thresholds of gestational BMI at 24 to 28 gestational weeks were defined by The HAPO cohort (16) accounting for gestational weight gain and pre-pregnancy BMI. Therefore, thresholds are appropriately higher than those for the non-pregnant adults. The smoking status was obtained from the questionnaires.

In addition, we also conducted two new CVR score models for sensitivity analysis. One was based on the five “clinical” CVR metrics (BMI, BP, triglyceride [TG] level, smoking status, and blood glucose level) and the other was based on the other metrics (pre-pregnancy BMI, BP, TC level, smoking status, and blood glucose level). The detailed classification criteria of TG and pre-pregnancy BMI are as follows: TG (mg/dl): ideal: <220, intermediate: 220–299, poor: ≥300. Pre-pregnancy BMI (kg/m^2^): ideal: ≤24.9, intermediate: 25–29.9, poor: ≥30 (17). The correlation coefficient among CVR score models were shown in the Supplementary Table 2.

### Laboratory analyses

The venous blood was collected from pregnant women at 16–23 gestational weeks. The blood samples were used to measure hypersensitive C-reactive protein, and 25(OH)D concentrations. The blood samples were centrifuged at 4°C and 2,056 × g for 5 min, quickly refrigerated at 4°C within 1 h, and then transferred to −80°C refrigerators within 8 h for long-term storage. The 25(OH)D and hs-CRP concentrations were determined using commercial chemiluminescence immunoassay kits (DiaSorin Stillwater, MN, United States) and turbidimetric inhibition immunoassay kits (Leadman biochemistry, Beijing, China) by well-trained researchers. The coefficient of variation (CV) between and within classes is less than 10%. Serum 25(OH)D concentrations were divided into two groups (<50 nmol/L and ≥50 nmol/L) (18).

## Statistical analysis

Demographic characteristics and clinic data were compared between different EDIP scores groups using the ANOVA for continuous variables and Chi-square analysis for the categorical variables. Variables were represented by the percentage or means (standard deviations, SDs).

Based on the restricted cubic spline hazard model, the association between EDIP score and increased CVR was shown. Based on the cubic curve-fitting models, the association of EDIP score with CVR score and hs-CRP or between hs-CRP and CVR score was shown.

Stratified analyses were used to estimate the association of EDIP scores with increased CVR according to serum 25(OH)D concentrations. We also conducted *post hoc* sensitivity analyses for the association between EDIP and gestational CVR based on the other CVR score models (included TG instead of TC or included pre-pregnancy instead of BMI at 24 to 28 gestational weeks). The analyses were performed using SPSS version 26.0 software (IBM Corp, Armonk, NY, United States). With a two-tailed *P*-value of <0.05 is considered significant.

## Results

Attrition analyses showed that the distributions of the sociodemographic characteristics, perinatal health status, and pregnancy lifestyle factors in nonparticipants did not differ from the participants. At the baseline, the average participant age was 29.1 (SD = 4.2) years, and the mean pre-pregnancy BMI was 21.5 (SD = 2.9) kg/m^2^. The proportion of women with increased CVR was 54.3%. Table 1 shows the baseline characteristics of the study participants according to the EDIP score. The education and sedentary time differed across 3 groups divided by the EDIP score (*P* < 0.05).

**TABLE 1:** Characteristics of the study population.

Characteristics	All (*n* = 3713)	EDIP score^1^			*P* value^2^
		Low (*n* = 929)	Intermediate (*n* = 1858)	High (*n* = 926)	
**CVR**					
Ideal BMI, n (%)	3412(91.9)	858(92.4)	1720(92.6)	834(90.2)	0.062
Ideal blood pressure, n (%)	3042(81.9)	781(84.1)	1521 (81.9)	740(79.9)	0.067
Ideal total cholesterol level, n (%)	2886(77.7)	730(78.6)	1449(77.9)	707(76.3)	0.478
Ideal glucose level, n (%)	3080(83.0)	787(84.7)	1544(83.1)	749(81.0)	0.088
Non-smokers, n (%)	3686(99.3)	922(99.2)	1847(99.4)	917(99.0)	0.536
CVR score, M ± SD	1.0 ± 1.1	0.9 ± 1.1	1.0 ± 1.1	1.0 ± 1.2	0.033
25(OH)D concentration, M ± SD, nmol/L	38.54 ± 16.31	39.45 ± 17.00	38.42 ± 16.41	37.90 ± 15.36	0.110
Hs-CRP concentration, M ± SD, mg/L	3.23 ± 2.34	3.02 ± 2.15	3.24 ± 2.36	3.44 ± 2.49	0.001
**Sociodemographic characteristics**					
Age, M ± SD, years	29.1 ± 4.2	29.1 ± 4.4	29.2 ± 4.1	29.1 ± 4.3	0.748
Urban residence, *n* (%)	3442(92.7)	861(92.7)	1727(92.9)	854(92.2)	0.786
Bachelor’s degree and above, *n* (%)	928(25.0)	192(20.7)	501(27.0)	235(25.4)	0.001
Household income >8000 yuan/m, *n* (%)	873(23.5)	231(24.9)	431(23.2)	211(22.8)	0.517
**Perinatal health status**					
Pre-pregnancy BMI, M ± SD, kg/m^2^	21.5 ± 2.9	21.5 ± 3.0	21.4 ± 2.8	21.6 ± 3.0	0.092
Primipara, *n* (%)	1380(37.2)	337(36.3)	685(36.9)	358(38.7)	0.529
Excessive GWG^3^, *n* (%)	1865(50.2)	481(51.8)	913(49.1)	471(50.9)	0.383
Family history of diabetes^4^, *n* (%)	336(9.1)	75(8.1)	180(9.7)	81(8.8)	0.350
Family history of hypertension^4^, *n* (%)	1241(33.4)	305(32.8)	628(33.8)	308(33.3)	0.871
**Pregnancy lifestyle factors, *n* (%)**					
Physical activity (≥ 3 days/week)	1683(45.3)	395(42.5)	861(46.3)	427(46.1)	0.138
Outdoor time (≥ 60 min/day)^5^	1155(31.1)	286(30.8)	588(31.6)	281(30.3)	0.760
Sedentary time (≥ 4 h/day)	2507(67.5)	597(64.3)	1254(67.5)	656(70.8)	0.010
Vitamin D supplementation≥ 3 days/week	2011(54.2)	501(53.9)	1018(54.8)	492(53.1)	0.701

BMI, Body Mass Index; EDIP, Empirical dietary inflammation pattern. GDM, gestational diabetes mellitus; GWG, gestational weight gain, CVR, cardiovascular risk.

^1^EDIP: Low, (< P_25_); Intermediate, (P_25_-P_75_); High, (≥ P_75_).

^2^P-value was from the analysis of variance (for means) or chi-square (for proportions).

^3^Excessive GWG: GWG > P_50_

^4^A family history of hypertension or diabetes was defined as either parent having hypertension or diabetes.

^5^Outdoor time means time spent outdoors in the daytime.

**FIGURE 1:**
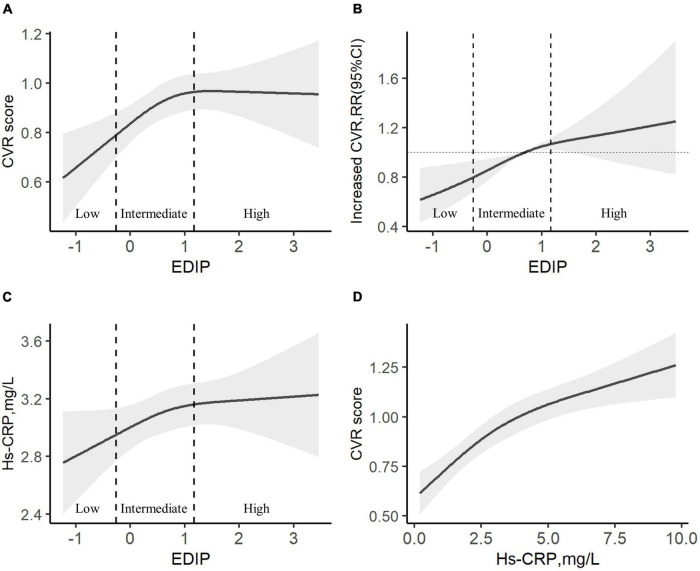
The association among EDIP scores, CVH, and hs-CRP. **(A)** A cubic curve-fitting model of the curvilinear association between EDIP and CVR score. **(B)** A restricted cubic spline hazard of the curvilinear association between EDIP and Increased CVR. **(C)** A cubic curve-fitting model of the curvilinear association between EDIP and hs-CRP. **(D)** A cubic curve-fitting model of the curvilinear association between hs-CRP and CVR score. All models were adjusted for age, residence, education, income, pre-pregnancy BMI, parity, gestational weight gain, family history of diabetes and hypertension, physical activity, outdoor time, sedentary time, and vitamin D supplementation frequency. Increased CVR, CVR score >0 points. CVR, cardiovascular risk, EDIP, Empirical dietary inflammation pattern.

In a cubic curve-fitting model fully adjusted for potential confounders, CVR score increased significantly with the increasing EDIP score in low and intermediate EDIP groups (Figure 1A). There was a significant positive association between the EDIP score and increased CVR (Figure 1B) or hs-CRP levels (Figure 1C). In addition, there was a significant positive association between hs-CRP levels and the CVR score (Figure 1D).

**FIGURE 2:**
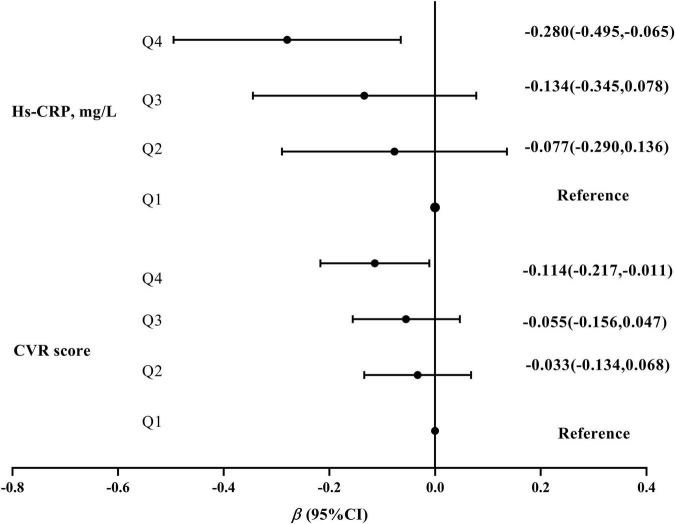
The association of 25(OH)D concentrations with CVR scores and hs-CRP levels. 25(OH)D concentrations were divided into four groups by the quartile (Q1\Q2\Q3\Q4). All models were based on the multiple linear regression and adjusted for age, residence, education, income, pre-pregnancy BMI, parity, gestational weight gain, family history of diabetes and hypertension, physical activity, outdoor time, sedentary time, and vitamin D supplementation frequency. CVR, cardiovascular risk.

In multiple linear regression models, the β (95% CI) of CVR score and hs-CRP levels were −0.114 (−0.217, −0.011) and −0.280 (−0.495, −0.065) in the highest quartile compared with the lowest quartile of 25(OH)D (*P* for trend of <0.05), respectively (Figure 2).

Table 2 compares the difference in 25(OH)D and hs-CRP levels and found that 25(OH)D concentrations were the lowest and hs-CRP levels were the highest in the high EDIP group (*P* for trend <0.05). The further stratified analysis found that 25(OH)D concentrations were the lowest and hs-CRP levels were the highest in the high EDIP group when 25(OH)D concentrations were <50 nmol/L. Increased CVR connected with high EDIP scores was observed only in women with 25(OH)D concentrations <50 nmol/L (RR = 1.85; 95% CI: 1.35∼2.54) (Table 2). Sensitivity analyses produced similar results (Supplementary Table 3).

**TABLE 2:** The association between EDIP and increased CVR stratified by vitamin D status.^1^

Groups	n (%)	25(OH)D^2^, nmol/L M ± SD	Hs-CRP^3^, mg/L M ± SD	Increased CVR^4^	
				n (%)	*RR*^1^ (95% *CI*)
**Overall**					
Low EDIP	929 (25.0)	39.45 ± 17.00	3.02 ± 2.15	465 (50.1)	1.00
Intermediate EDIP	1858(50.0)	38.42 ± 16.41	3.24 ± 2.36	1025(55.2)	**1.23 (1.05, 1.44)**
High EDIP	926 (25.0)	37.90 ± 15.36	3.44 ± 2.49	525 (56.7)	**1.31 (1.09, 1.58)**
**25(OH)D ≥ 50 nmol/L**					
Low EDIP	200 (25.0)	64.78 ± 15.16	2.64 ± 1.96	87 (43.5)	1.00
Intermediate EDIP	400(49.9)	62.44 ± 13.59	3.16 ± 2.28	203 (50.7)	1.33 (0.94, 1.88)
High EDIP	201 (25.1)	60.64 ± 8.44	3.31 ± 2.21	101 (50.2)	1.33 (0.90, 1.98)
**25(OH)D < 50 nmol/L**					
Low EDIP	733 (25.2)	32.50 ± 9.19	3.12 ± 2.20	380 (51.8)	**1.41 (1.03, 1.94)**
Intermediate EDIP	1458(50.1)	31.83 ± 9.53	3.27 ± 2.38	822 (56.4)	**1.70 (1.26, 2.29)**
High EDIP	721 (24.7)	31.57 ± 9.72	3.49 ± 2.51	422 (58.5)	**1.85 (1.35, 2.54)**

CVR, cardiovascular risk, EDIP, Empirical dietary inflammation pattern.

^1^These models were adjusted for age, residence, education, income, pre-pregnancy BMI, parity, gestational weight gain, family history of diabetes and hypertension, physical activity, outdoor time, sedentary time, and vitamin D supplementation frequency.

^2^P for trend across 3 groups (EDIP) was 0.041, P for trend across 6 groups [EDIP * 25(OH)D concentrations] was <0.001.

^3^P for trend across 3 groups (EDIP) was < 0.001, P for trend across 6 groups [EDIP * 25(OH)D concentrations] was <0.001. Increased CVR, (cardiovascular risk score >0 point).

**FIGURE 3:**
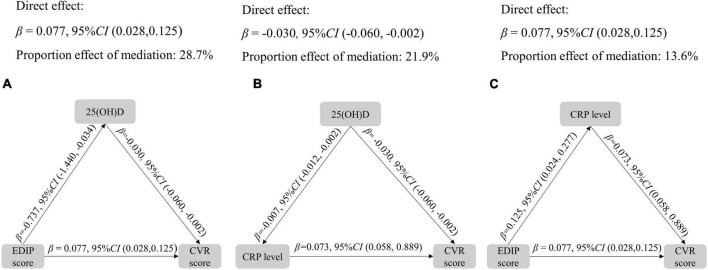
Mediation analysis among EDIP score, CVR score, and hs-CRP. All models adjusted for age, residence, education, income, pre-pregnancy BMI, parity, gestational weight gain, family history of diabetes and hypertension, physical activity, outdoor time, sedentary time, and vitamin D supplementation frequency. CVR, cardiovascular risk, EDIP, Empirical dietary inflammation pattern.

The role of hs-CRP and 25(OH)D in the association between EDIP score and CVR score were evaluated by the structural equation models. As shown in Figure 3, mediation analysis revealed that the proportion of association between the EDIP score and CVR score mediated by 25(OH)D was 28.7%. The proportion of the association between 25(OH)D concentrations and the CVR score mediated by hs-CRP was 21.9%. In addition, the proportion of the association between the EDIP score and the CVR score mediated by hs-CRP was 13.6%.

## Discussion

To our knowledge, this is the first study to evaluate the role of vitamin D status in the association between dietary inflammatory potential and gestational CVH. We observed that the EDIP score was positively associated with the CVR score in a dose-response fashion, independent of traditional risk factors. We also found that the association was significantly modified by serum 25(OH)D concentrations, while the association between high EDIP scores and increased CVR appeared to be attenuated among the participants with sufficient serum 25(OH)D concentrations.

The relationship between dietary inflammation and CVH has been gradually recognized in recent years. An anti-inflammatory diet, rich in fiber, antioxidants, and long-chain-3 polyunsaturated fatty acids, may positively impact CVH (19, 20). A randomized controlled trial also showed that adherence to a Mediterranean diet (MeDiet) could reduce the incidence of cardiovascular disease by 30%, compared with the control diet (21). Conversely, a prospective study did not support the protective effect of high-dietary antioxidant levels on CVH (22). Another multicenter randomized study trial also found that the MeDiet in pregnancy did not reduce CVD risk (23). Conclusions based on these investigations were inconsistent, the leading cause may be the dietary indices such as Alternate Mediterranean Diet, Dietary Approaches to Stop Hypertension (DASH), and Alternative Healthy Eating Index, which generally assessed the whole dietary quality rather than dietary inflammatory potential. Notably, we used a diet index EDIP, which strengthened the evaluation of dietary inflammatory potential. EDIP shares only a few foods with other dietary indexes (thus explaining its moderate correlation) and emphasizes unique inflammation-related foods. In addition, our findings are consistent with the Nurses’ Health Study (NHS) cohort that a higher dietary inflammatory potential, as revealed by the higher EDIP scores, was associated with an increased risk of CVD (5). Our findings also found that a systemic inflammatory marker (hs-CRP) played a mediating role in such association. In a study of diet interventions to prevent CVD, high-inflammation marker levels moderated the effects of the DASH (7). Accordingly, decreased inflammation may lead to consequently improved gestational CVH. Thus, interventions to reduce inflammation and thus protect CVH applicable to the pregnant women are needed, and vitamin D supplements are an attractive target.

Vitamin D is an everyday nutritional supplement during pregnancy and may exhibit several anti-inflammatory effects (24, 25). In this study, our findings showed adequate 25(OH)D concentrations were associated with lower hs-CRP levels. In addition, we found that adequate 25(OH)D concentrations may modify gestational CVH by influencing hs-CRP levels. We also found that the inverse association between 25(OH)D concentrations and the CVR score could be mediated by hs-CRP levels. Recent evidence shows that serum 25(OH)D concentrations are negatively correlated with systemic inflammatory markers such as CRP (25). The previous study also found that high-serum 25(OH)D concentrations may reduce CVD risk through modulation of inflammatory processes, which was similar to our study (26). In the present study, we also found that there was no significant association between high EDIP and increased CVR among participants with sufficient serum 25(OH)D concentrations. On the one hand, this modification may be through the anti-inflammatory effects of vitamin D. On the other hand, 25(OH)D concentrations may also directly mediate the association between high-EDIP scores and increased gestational CVR, which is also confirmed by our results.

In addition, this study found that vitamin D deficiency is common during pregnancy, and 78.4% of women had 25(OH)D concentrations <50 nmol/L. However, the majority of developing nations, including China, do not offer vitamin D deficiency screening during pregnancy, and most pregnant women also do not follow the recommendation regarding vitamin D supplementation. Our study suggests that vitamin D supplementation during pregnancy may have potential benefits on the gestational CVH.

The mechanisms underlying the vitamin D effect on the association between inflammatory dietary patterns and CVD risk remain unclear. Several potential mechanisms may explain the relations. For example, 25(OH)D_3_ as an anti-inflammatory compound can inhibit nuclear factor kappa beta (NF–κB) activation through increased vitamin D receptor (VDR) expression. So, vitamin D deficiency can induce inflammation of the blood vessel walls and promote atherosclerosis by enhancing NF–κB activation (27). In addition, vitamin D deficiency can increase inflammation, enhance inflammatory cytokines expression, and inhibit VDR expression and activity. This may lead to enhanced signaling of downstream inflammatory signaling cascades resulting in various CVD (28). A previous study suggests that high 25(OH)D concentrations may reduce CVD risk by modulating immune function and inflammatory processes (26). In addition, laboratory and animal study data indicated that 25(OH)D inhibits vascular smooth muscle cell proliferation and vascular calcification, controls volume homeostasis and blood pressure *via* regulation of the renin-angiotensin-aldosterone system and exerts anti-inflammatory effects (29–31). These findings indicate that vitamin D regulates blood pressure by acting on the endothelial and smooth muscle cells and thus plays an essential anti-inflammatory role in CVH. These anti-inflammatory effects of vitamin D may modify the association between a high EDIP score and increased gestational CVR.

This is the first study examining the moderating effect of vitamin D on the relationship between a pro-inflammatory diet and gestational CVH. In addition, the EDIP, a validated, empirically developed, food-based tool, was used to strongly assess the dietary inflammation potential. Although a single inflammation biomarker was measured in this study, the significant correlation between hs-CRP levels and the EDIP score supports the validity of EDIP evaluation. To sum up, we adjusted for broad sociodemographic characteristics; the sample size was relatively large and reduced residual confounding.

### Study limitations

First, our research cannot draw causality, and it takes longer to verify cardiovascular events. In addition, our findings need to be confirmed in the randomized clinical trials. Second, self-reported FFQ diet data of the pregnant women may have measurement errors, which usually weakens the actual connection. Third, we did not consider the effect of the participants’ salt intake on CVH. Furthermore, the data on CVR was not collected at the baseline, and we are not able to assess the CVR status at the baseline of the included individuals. Hence, the temporality and the causality between diet and CVR are compromised in this study. In addition, only hs-CRP was measured for inflammation biomarkers. Hence, inflammatory status of individuals cannot be evaluated comprehensively. Moreover, a caution should be taken when interpreting this study results, since previous studies (32–37) have shown that components of the CVR score in this study (BMI, blood pressure, total cholesterol, glucose levels, and smoking status, and also triglyceride levels) are in inverse association with vitamin D levels, and therefore, a higher CVR score should be automatically associated with lower vitamin D levels in our study. To sum, our research was conducted only on the pregnant Chinese women. Therefore, our research results may need to be extended to other populations for verification.

## Conclusion

In sum, our research indicates that the regulation of chronic inflammation may be a potential mechanism linking dietary patterns and gestational CVR, and vitamin D may have anti-inflammatory effects to reduce cardiovascular risk caused by the pro-inflammatory foods. Reducing the inflammation potential of the diet among pregnant women may provide an effective strategy for promoting CVH. Future studies need to verify the potential protective effects of vitamin D supplementation during pregnancy on cardiovascular health induced by a pro-inflammatory diet.

## Data availability statement

The original contributions presented in the study are included in the article/Supplementary material, further inquiries can be directed to the corresponding author.

## Ethics statement

The studies involving human participants were reviewed and approved by the Ethics Committee of Anhui Medical University (No. 20180092). The patients/participants provided their written informed consent to participate in this study.

## Author contributions

W-JY performed the experiments and was responsible for the collection and compilation of data, analysis of data, and writing the manuscript. L-JY contributed to the compilation of the data and helped wrote the manuscript. LW, F-CD, QL, and LZ were responsible for collecting clinical data and contributing to clinical assessments. R-XT and X-MJ designed the study and assisted with the data collection. PZ was the guarantor of this work designed and supervised the study and revised the manuscript. All authors read and approved the final manuscript.
