# Supplementary material for: Adequate 25(OH)D moderates the relationship between dietary inflammatory potential and cardiovascular health risk during the second trimester of pregnancy
Source: Front Nutr. 2022 Jul 29;9:952652. doi: 10.3389/fnut.2022.952652 (PMC9372498; doi:10.3389/fnut.2022.952652)
Supplement: Supplementary file 1 [file Data_Sheet_1.pdf]

Supplementary Table 1 Food groups used to calculate the Empirical Dietary Inflammatory Pattern (EDIP) score[1]

Food groups	Food items	Weights[2]
Pro-inflammatory		
Processed meat	Sausages, bacon, hot dog, marinated pork	165.03
Red meat	Beef, lamb, pork	140.19
Organ meat	Livers	144.61
Other fish	Canned tuna, shrimp, lobster, scallops, fish, or other seafood	252.45
Other vegetables	Corn, mushrooms, green /red peppers, cucumbers	136.14
Refined grains	White bread, roll bread, white rice, biscuits, noodle, dumplings,	81.21
High energy beverages	Soft drinks, other carbonated beverages with sugar, or fruit punch drinks	156.85
Low-energy beverages	Low-energy cola; other low-energy carbonated beverages	94.77
Tomatoes	Fresh tomato, tomato juice, or tomato sauce	167.92
Anti-inflammatory		
Beer	Beer	-136.99
Wine	Wine	-249.70
Coffee	Coffee	-42.25
Tea	Tea	-83.18
Dark yellow vegetables	Carrots, sweet potatoes, winter squash	-165.37
Leafy green vegetables	Spinach, Chinese cabbage	-190.29
Snacks	Potato/corn chips, popcorn, crackers	-45.08
Fruit Juice	Apple juice, orange juice, grape juice, prune juice, and other juice	-58.95

[1] The EDIP was calculated as the weighted sum of these 18 food groups with weights (i.e., the contributions of each food to the overall score) equal to the coefficients from the stepwise regression.

[2] Values are regression coefficients for each EDIP score component obtained from the last step of the stepwise linear regression analysis

Supplementary Table 2 Correlations of different CVR score models

CVR score[1]	CVR score		
	Model 1	Model 2	Model 3
Model 1	1.00	0.910	0.933
Model 2		1.000	0.846
Model 3			1.000

Correlations were calculated using the Pearsons correlation coefficient. CVR, cardiovascular risk

[1] Model 1 consists of BMI, BP, TC level, smoking status, and blood glucose level; Model 2 consists of BMI, BP, triglyceride level, smoking status, and blood glucose level; Model 3 consists of pre-pregnancy BMI, BP, TC level, smoking status, and blood glucose level.

Supplementary Table 3 Association between EDIP and Increased CVR

EDIP score	Increased CVR [1]		
	Model 1	Model 2	Model 3
	RR[2] (95% *CI*)	*RR*[2] (95% *CI*)	*RR*[2] (95% *CI*)
Low EDIP	1.00	1.00	1.00
Intermediate EDIP	**1.23 (1.05, 1.44)**	**1.26 (1.08, 1.48)**	**1.20 (1.02, 1.41)**
High EDIP	**1.31 (1.09, 1.58)**	**1.31 (1.09, 1.57)**	**1.27 (1.05, 1.52)**

CVR, cardiovascular risk, EDIP, Empirical dietary inflammation pattern.

[1] Adjusted for age, residence, education, income, pre-pregnancy BMI, parity, gestational weight gain, family history of diabetes and hypertension, physical activity, outdoor time, sedentary time, and vitamin D supplementation frequency.

[2] Model 1 consists of BMI, BP, TC level, smoking status, and blood glucose level; Model 2 consists of BMI, BP, triglyceride level, smoking status, and blood glucose level; Model 3 consists of pre-pregnancy BMI, BP, TC level, smoking status, and blood glucose level.

Online Supporting Material Page 4

Supplemental Figure 1

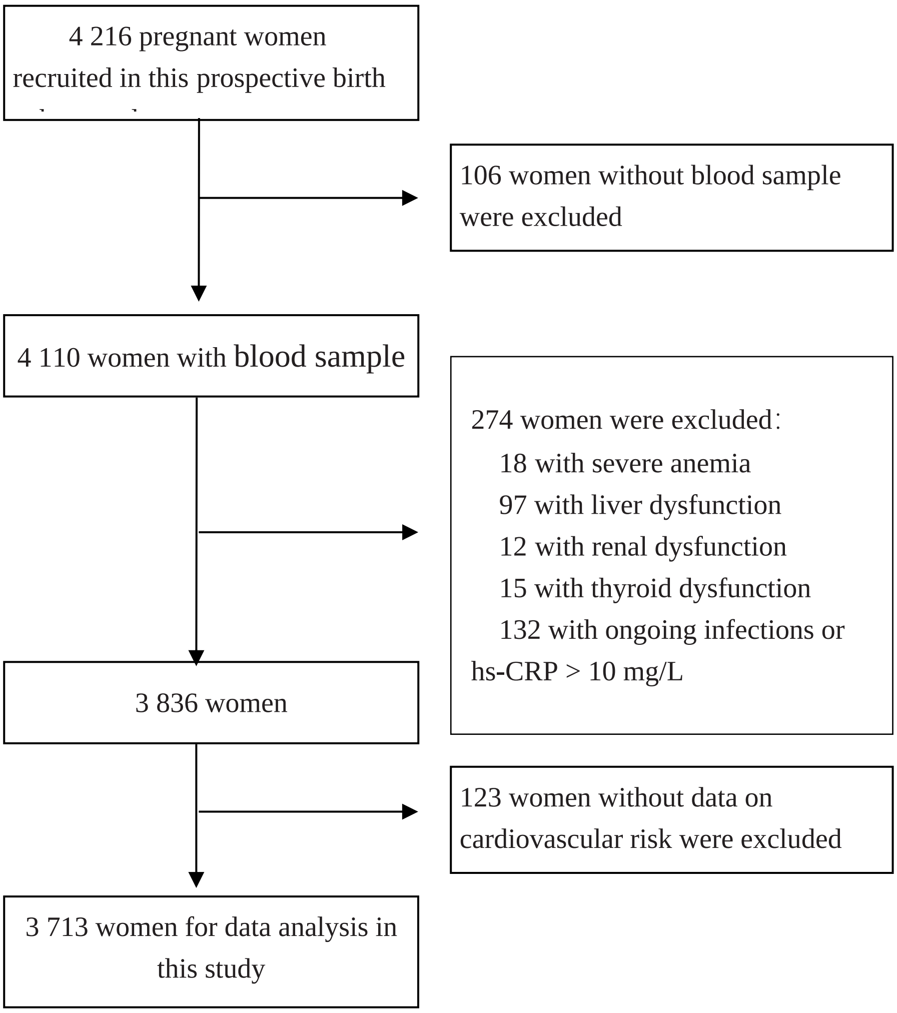

Supplementary Figure 1 Participants flow chart
